# Preparative Isolation and Purification of Five Flavonoid Glycosides and One Benzophenone Galloyl Glycoside from *Psidium guajava* by High-Speed Counter-Current Chromatography (HSCCC)

**DOI:** 10.3390/molecules181215648

**Published:** 2013-12-16

**Authors:** Yindi Zhu, Yue Liu, Ying Zhan, Lin Liu, Yajuan Xu, Tunhai Xu, Tonghua Liu

**Affiliations:** 1 Department of Traditional Chinese Medicine Chemistry, School of Traditional Chinese Medicine, Beijing University of Chinese Medicine, No. 6 Wangjing Zhonghuan South Road, Chaoyang District, Beijing 100102, China; E-Mails: zhuyindi314@sina.com (Y.Z.); zhanying0918@163.com (Y.Z.); lyn9500@163.com (L.L.); 2 Health-cultivation Laboratory of the Ministry Education, Beijing University of Chinese Medicine, 11 North Third Ring Road East, Chaoyang District, Beijing 100029, China; E-Mail: thliu@tom.com; 3 Chemistry of Chinese Medicine, Jilin Academy of Chinese Medicine Sciences, Changchun 130021, China; E-Mails: ydzh33@126.com (Y.L.); xyj6492@sohu.com (Y.X.)

**Keywords:** HSCCC, flavonoid glycosides, benzophenone galloyl glycoside, *Psidium guajava*

## Abstract

*Psidium guajava* leaves have a diverse phytochemical composition including flavonoids, phenolics, meroterpenoids and triterpenes, responsible for the biological activities of the medicinal parts. In particular, flavonol glycosides show beneficial effects on type II diabetes mellitus. A simple and efficient HSCCC method has been developed for the preparative separation of five flavonoid glycosides and one diphenylmethane glycoside from *P. guajava*. A solvent system composed of *n*-hexane–ethyl acetate–methanol–water (0.7:4:0.8:4, v/v/v/v) was optimized for the separation. The upper phase was used as the stationary phase, and the lower phase was used as the mobile phase. Under the optimized conditions, hyperoside (15.3 mg), isoquercitrin (21.1 mg), reynoutrin (65.2 mg), quercetin-3-*O-β*-D-arabinopyranoside (71.7 mg), quercetin-3-*O-α*-L-arabinofuranoside (105.6 mg) and 2,4,6-trihydroxy-3,5-dimethylbenzophenone 4-*O*-(6''-*O*-galloyl)-*β*-D-glucopyranoside (98.4 mg) were separated from crude sample (19.8 g). The structures of all the isolates were identified by ESI-MS, ^1^H- and ^13^C-NMR analyses and their purities (>95%) were determined using HPLC.

## 1. Introduction

*Psidium guajava* (guava), an important food crop and medicinal plant in tropical and subtropical countries, is widely used as a food and in folk medicine around the World. It is a native to Mexico and extends throughout South America, Europe, Africa and Asia [1,2]. Guava fruits are beneficial for health [3], protecting kidney against diabetic progression via their anti-oxidative, anti-inflammatory and anti-glycative effects [4]. Guava leaves are used for the treatments of type II diabetes mellitus [5] and diarrhoea [6], showing hypoglycemic [7,8], antioxidant [9,10], anti-inflammatory [11], anticancer [12] gastro protective [13] and antipathogenic microorganism effects [14].

Previous phytochemical studies have demonstrated that phenolics [15], flavonoids [16], meroterpenoids [17,18] and triterpenes [19] were the major bioactive constituents of *P. guajava* leaves. Flavonol glycosides of guava have beneficial effects for type II diabetes mellitus by inhibiting dipeptidyl peptidase activity [20], and adipogenesis in 3T3-L1 preadipocytes via down-regulation of PPARg and C/EBP a expression [21]. Hyperoside has a great number of biological effects, such as anti-inflammatory, anticancer and antioxidant activities [22], quercetin, an antioxidant, has a preventive effect on thioacetamide-induced liver cell necrosis [23], reynoutrin showed a-glucosidase inhibitory activity [24], quercetin-3-*O-β*-d-arabinopyranoside and quercetin-3-*O-α*-l-arabinofuranoside possess antibacterial and antifungal activities [25] and 2,4,6-trihydroxy-3,5-dimethylbenzophenone 4-*O*-(6''-*O*-galloyl)-*β*-d-glucopyranoside showed significant inhibitory activities against histamine release from rat peritoneal mast cells [26].

Traditional isolation techniques for these components have required the use of multiple steps employing column chromatography, medium-pressure liquid chromatography, vacuum column chromatography, preparative HPLC, or combinations of these techniques [18]. However, high-speed counter-current chromatography, which is free of irreversible adsorption [27], is capable of isolating multiple components from plant extracts, with added benefits over traditional methods, including no losses and lower solvent use and has been widely used in preparative separation of natural product [28,29].

**Figure 1:**
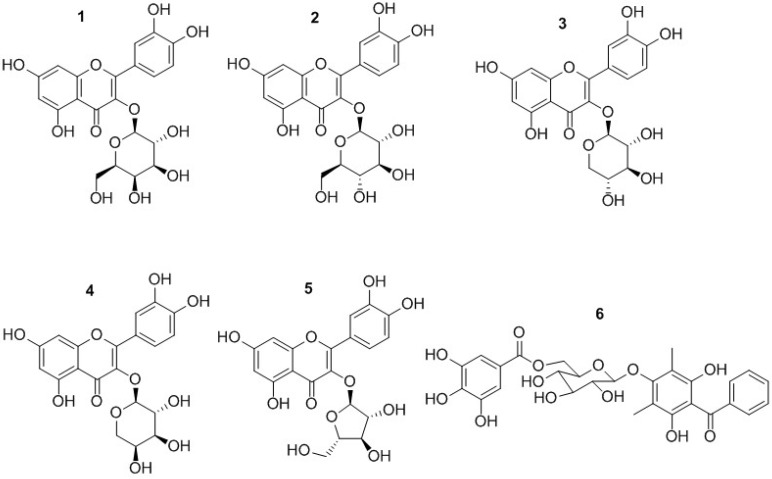
Chemical structures of five flavonoid glycosides and one benzophenone galloyl glycoside from of *P. guajava* leaves: quercetin-3-*O-β*-D-galactopyranoside (**1**), quercetin-3-*O-β*-D-glucopyranoside (**2**), quercetin-3-*O-β*-D-xylopyranoside (**3**), quercetin-3-*O-β*-D-arabinopyranoside (**4**), quercetin-3-*O-α*-L-arabinofuranoside (**5**), 2,4,6-trihydroxy-3,5-dimethylbenzophenone 4-*O*-(6''-*O*-galloyl)-*β*-D-glucopyranoside (**6**).

In this paper, we report a new method using HSCCC and semi-preparative HPLC for the isolation and purification of five flavanoids and one benzophenone galloyl glycoside (Figure 1) from *P. guajava* leaves, four of which were purified using only the HSCCC separation, while a hyperoside and isoquercitrin mixture was separated by semi-preparative liquid chromatography.

## 2. Results and Discussion

### 2.1. Solvent System Selection

A suitable solvent system plays an important role in separation by HSCCC. The system in which the main compounds could partition in the two phases with partition coefficients about 0.5–5 was used for HSCCC separation [30]. A smaller *K* value elutes the solute closer to the solvent front with lower resolution while a larger *K* value tends to give better resolution but broader, more dilute peaks due to a longer elution time. In this experiment, the ethyl acetate extract was analyzed by HPLC-UV and 6 major unknown peaks were targeted for further separation by HSCCC. Two-phase solvent systems with *n*-hexane–ethyl acetate–methanol–water (1:1:1:1, 1:2:1:2, 1:3:1:3, 1:4:1:4, 1:5:1:5, 1:6:1:6, 0.7:4:0.8:4, 0.3:3:0.1:3) were tested according to the polarity of the target compounds, and the results are shown in Table 1.

When the target analyte is unknown, the search may start at *n*-hexane–ethyl acetate–methanol–water (1:1:1:1, v/v) and then follow the direction indicated by *K* values [31]. The first solvent system of *n*-hexane–ethyl acetate–methanol–water (1:1:1:1) was tested, and the analytes were eluted close to the solvent front with poor separation, whereas a gradual decrease in the volume ratio of *n*-hexane and water yielded a better resolution. Finally, good separation results of the ethyl acetate extract were obtained using the solvent system of *n*-hexane–ethyl acetate–methanol–water (0.7:4:0.8:4). A solvent system consisting of *n*-hexane–ethyl acetate–methanol–water (0.3:3:0.1:3, v/v/v/v) was used for the purification of mixture of **1** (*K* = 1.27) and **2** (*K* = 1.19). A solvent system consisting of *n*-hexane–ethyl acetate–methanol–water (0.7:4:0.8:4) was used for the purification of compound **3** (*K* = 1.28), **4** (*K* = 1.68) and **5** (*K* = 2.64). A solvent system consisting of *n*-hexane–ethyl acetate–methanol–water (1:3:1:3) was used for the purification of **6** (*K* = 1.06).

**Table 1:** *K* values of compounds **1**–**6** from *P. guajava* leaves in two-phase solvent systems (*n*-hexane–ethyl acetate–methanol–water) for HSCCC separation.

Solvent Ratio	Partition Coefficient (*K*_U/L_)					
	1	2	3	4	5	6
1:1:1:1	0.27	0.30	0.09	0.06	0.07	0.08
1:2:1:2	0.09	0.11	0.18	0.24	0.32	0.41
1:3:1:3	0.14	0.17	0.33	0.46	0.65	1.06
1:4:1:4	0.40	0.43	1.07	1.48	2.28	3.94
1:5:1:5	0.32	0.34	0.88	1.25	1.90	3.66
1:6:1:6	0.48	0.51	1.47	2.03	3.43	6.57
0.7:4:0.8:4	0.50	0.53	1.28	1.68	2.64	4.40
0.3:3:0.1:3	1.27	1.19	4.10	5.67	11.03	26.64

### 2.2. HSCCC Separation

**Figure 2:**
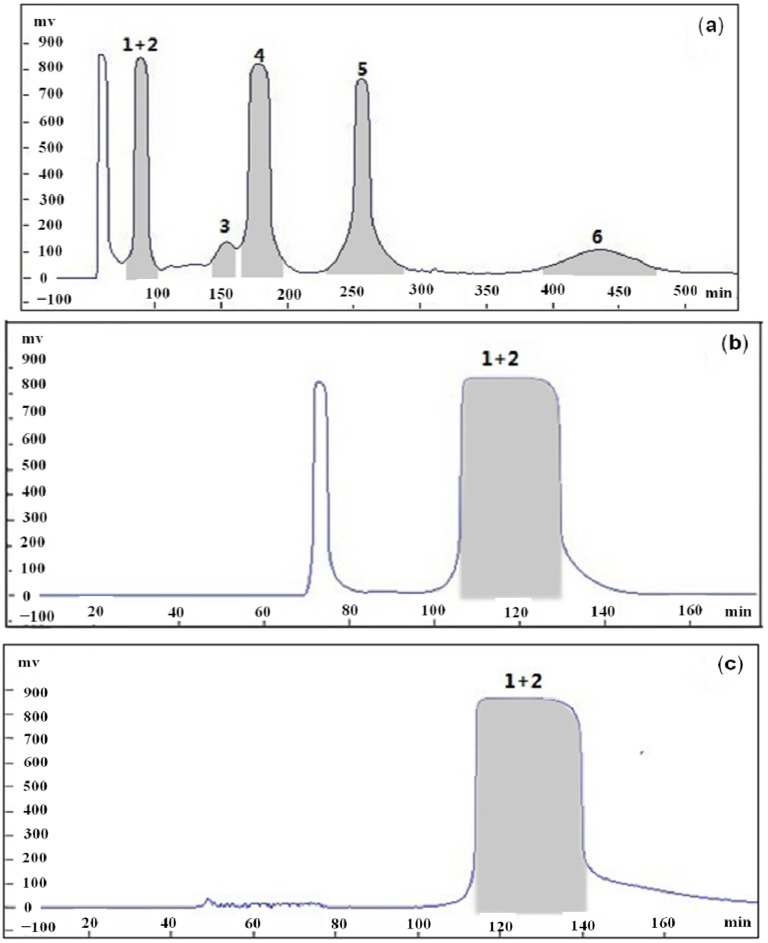
HSCCC chromatogram of the ethyl acetate extract from *P. guajava* leaves. (**a**) Ethyl acetate extract from *P. guajava* leaves, (**b**) first time purification of mixture of compound 1 and compound 2 and (**c**) second time purification of mixture of compound 1 and compound 2.

Under the optimized conditions, the crude extract (600 mg) was subjected to HSCCC, and the resulting HSCCC chromatogram is shown in Figure 2.

**Figure 3:**
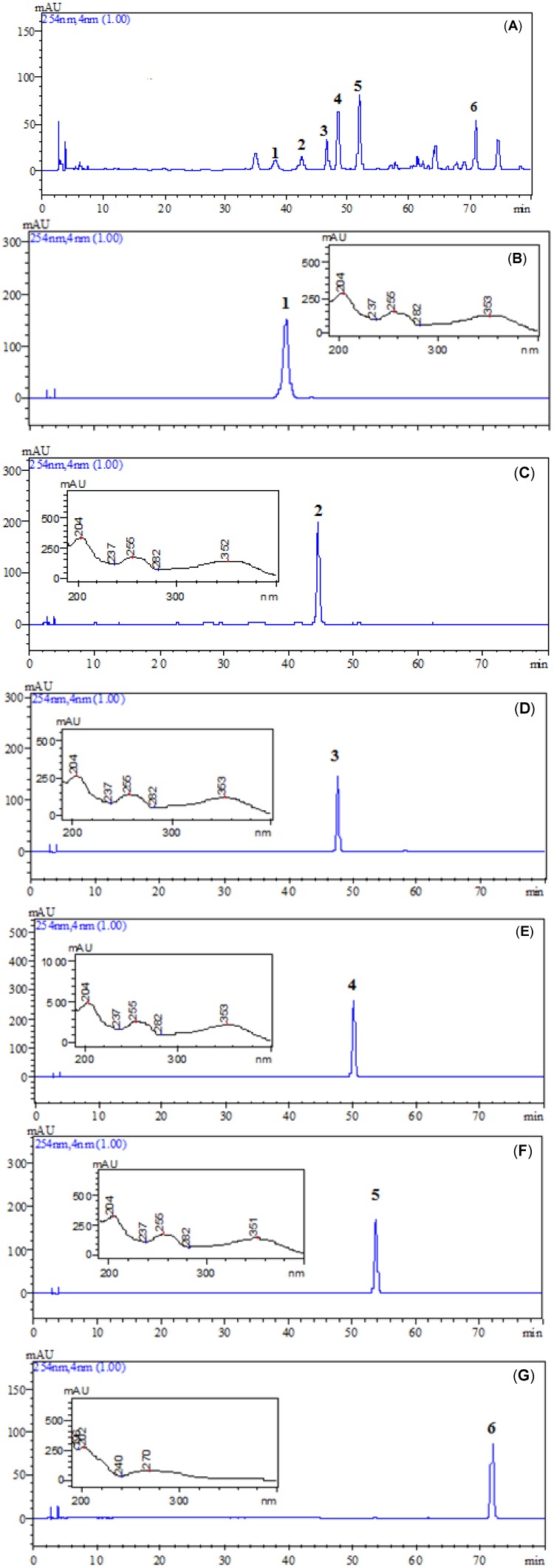
HPLC chromatograms of ethyl acetate extract from *P. guajava* leaves and HSCCC peak fractions. (**A**) Ethyl acetate extract from *P. guajava* leaves, (**B**–**C**) the two targeted compounds (compounds **1** and **2**) purified by HSCCC. (**D**–**G**) the four targeted compounds (compound **3**–**6**) purified by HSCCC.

Five fractions were obtained, each fraction was further purified by HSCCC, and then mixture of compounds **1** and **2** were separated by semi-preparative HPLC, resulting in the isolation of six compounds: **1** (15.32 mg), **2** (21.12 mg), **3** (65.15 mg), **4** (71.69 mg), **5** (105.57 mg) and **6** (98.43 mg). The crude sample and peak fractions separated by HSCCC and semi-preparative HPLC were analyzed by HPLC, under the optimum analytical conditions, and the chromatograms are presented in Figure 3.

As shown in Figure 3B–G, the HPLC analysis of each of the HSCCC fractions revealed that the purities of these six compounds were 98.1%, 98.9%, 95.4%, 99.5%, 99.7% and 98.4%, respectively. In order to save solvents and time, the compounds with higher *K* values, which were still retained in the column after compound **6** was eluted, were removed by forcing out the stationary phase by air compressor. After each run, the column was cleaned with 100 mL of ethanol.

## 3. Experimental

### 3.1. Materials and Reagents

The dried leaves of *P. guajava* were purchased from Beijing Twinbridge Yanjing Chinese Medicine Yinpian Factory (Beijing, China) in 2011. Effective plant leaves identification was provided by Professor Chunsheng Liu of the College of Traditional Chinese Medicine, Beijing University of Chinese Medicine, and the voucher specimen (201103012) was deposited in the Health-cultivation Laboratory of the Ministry of Education, Beijing, China. All organic solvents used for sample preparation and HSCCC were of analytical grade and purchased from the Beijing Chemical Works (Beijing, China). Methanol used for HPLC analysis was of chromatographic grade, and was purchased from Thermo Fisher Scientific Inc. (Waltham, MA, USA), and water (18.2 MΩ) was produced by a Milli-Q system (Millipore, Bedford, MA, USA). They were filtered and degassed prior to use.

### 3.2. Apparatus

A TBE-300B high-speed counter-current chromatography instrument (Shanghai Tauto Biotech Co., Ltd, Shanghai, China) with three polytetrafluoroethylene (PTFE) preparative coils (total volume: 340 mL) and a 20 mL sample loop were used for HSCCC. Solvents were delivered by a TBP 5002 (Shanghai Tauto Biotech Co., Ltd, Shanghai, China) pump. The UV-absorbance of the eluent was monitored by a TBD 2000 UV detector (Shanghai Tauto Biotech Co., Ltd, Shanghai, China) at the wavelength of 254 nm. A DC-0506 constant-temperature circulating implement (Shanghai Sunny Hengping Scientific Instrument Co., Ltd, Shanghai, China) was used to control the separation temperature. The data were collected using a HW-2000 chromatography workstation (Shanghai Qianpu Software Ltd., Shanghai, China). A FB-36/7 air compressor pump was used to force out the stationary phase. The analytical HPLC equipment was a LC-15C equipped with a SPD-M20A diode array detector, and a LCsolution chromatography workstation (Shimadzu, Kyoto, Japan). Semi-preparative HPLC was performed on a CXTH LC-3000 HPLC system with a CXTH LC-3000 UV spectrophotometric detector (Beijing Chuangxintongheng Science and Technology Co., Ltd., Beijing, China). The nuclear magnetic resonance (NMR) spectrometer was a Bruker AVANCE 600 MHz NMR (Bruker Spectrospin, Fällanden, Switzerland) with tetramethylsilane (TMS) as the internal standard.

**Figure 4:**
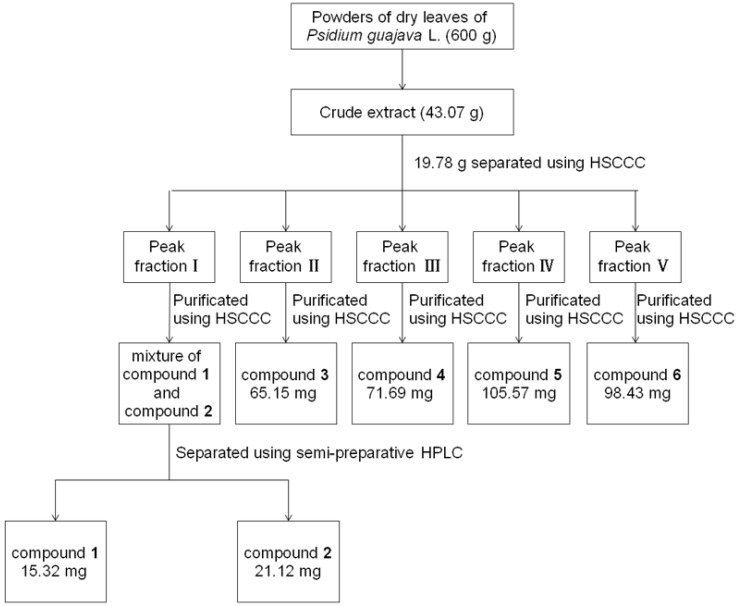
The sample preparation scheme.

### 3.3. Preparation of the Crude Sample

The dried leaves (600 g) of *P. guajava* was crushed with a grinder (Tianjin Taisite Instrument Co., LTD., Tianjin, China) and ultrasonically extracted seven times using ethyl acetate, each time with 2.5 L for 1 h. Then the extract was filtered and evaporated to dryness by rotary evaporation at 40 °C under reduced pressure, and dried in a vacuum oven at 60 °C for 24 h (43.05 g) and part of the dried extract (19.78 g) were prepared for subsequent HSCCC isolation and purification. The sample preparation scheme is shown in Figure 4.

### 3.4. Selection of the Solvent System

The partition coefficient is the ratio of the solute distributed between the mutually equilibrated two solvent phases. The partition coefficients were determined by HPLC as follows. A suitable amount of crude sample was added into a series of pre-equilibrated two-phase solvent systems, and the solution was then shaken fully. Subsequently, the same volume of each of upper and lower phases was evaporated to dryness. The residues were diluted into 2 mL methanol and then analysed by HPLC. The *K* value was defined as the peak area of the component in the upper phase divided by the peak area of the component in the lower phase.

### 3.5. Preparation of the Two-Phase Solvent Systems and Sample Solution

A solvent system consisting of *n*-hexane–ethyl acetate–methanol–water (0.7:4:0.8:4, v/v/v/v) was used for the separation of the ethyl acetate extract. A solvent system consisting of *n*-hexane–ethyl acetate–methanol–water (0.3:3:0.1:3, v/v/v/v) was used for the purification of mixture of compound **1** and compound **2**. A solvent system consisting of *n*-hexane–ethyl acetate–methanol–water (0.7:4:0.8:4, v/v/v/v) was used for the purification of compounds **3**, **4** and **5**. A solvent system consisting of *n*-hexane–ethyl acetate–methanol–water (1:3:1:3, v/v/v/v) was used for the purification of compound **6**. All solvent systems were thoroughly mixed, vented in a separatory funnel at room temperature and allowed to separate into two distinct phases before use. The sample solution was prepared by dissolving 600 mg of crude extract in 14 mL mixture of lower phase and upper phase (1:1, v/v) of the solvent system used for HSCCC separation.

### 3.6. Separation and Purification by HSCCC

The coiled column was filled with the upper phase. After the column was entirely filled, the apparatus was rotated at 900 rpm, while the lower phase (mobile phase) was pumped into the column at a flow rate of 2.5 mL/min. After hydrodynamic equilibrium was reached, as indicated by the emergence of the mobile phase front, a 14 mL sample solution containing 600 mg of the crude powder was injected into the column through the injection valve. The effluent from the tail end of the column was continuously monitored with a UV detector at 254 nm, and the chromatogram was recorded. The temperature of the apparatus was set to 25 °C. The peak fractions were collected manually according to the elution profile and evaporated under reduced pressure, and the residues were dissolved in methanol for subsequent purity analysis by HPLC. The purity was obtained by HPLC peak area calculation.

### 3.7. Separation of Mixture of Compound **1** and Compound **2** by Semi-Preparative HPLC

The mixture of compounds **1** and **2** was separated by semi-preparative HPLC performed with a Thermo Scientific Hypersil Gold Phenyl column (5 μm, 10 × 250 mm). The mobile phase solvents consisted of water as eluent A and acetonitrileas eluent B (87:13, v/v). The flow-rate was 2.0 mL/min and 1.0 mL of sample solution (1 mg/mL of fraction I from HSCCC separation in methanol) was injected through the sample injector. The effluent from the outlet of the column was monitored at 254 nm. The peak fractions were collected according to the chromatogram.

### 3.8. HPLC Analysis

The crude extract and each peak fraction obtained by HSCCC were analysed by HPLC. The HPLC analyses were carried out using a Thermo Scientific Syncronis C18 column (250 × 4.6 mm ID, 5μm). The HPLC solvents were H_2_O with 0.2% phosphoric acid (v/v) as an aqueous solvent (A) and CH_3_CN as an organic solvent (B). The gradient condition was as follows: 0–35 min, 14% B; 35–40 min, 14%–18% B; 40–50 min, 18% B, 50–55 min, 18%–25% B; 55–75 min, 25% B. A flow rate was set at 1.0 mL/min, a column temperature was 40 °C and sample injection volume was 20 µL.

### 3.9. Identification of Target Compounds

The chemical structures of peak fractions separated by HSCCC were identified according to their ESI-MS, ^1^H-NMR and ^13^C-NMR data. The data of each peak fraction are provided below.

*Compound* **1**. Light yellow powder, ESI-MS (+ve ion mode): *m/z* 465.1 [M+H]^+^, 487.1 [M+Na]^+^; ^1^H-NMR (DMSO-*d*_6_) *δ*: 6.20 (1H, d, *J* = 1.8 Hz, H-6), 6.40 (1H, d, *J* = 1.8 Hz, H-8), 7.53 (1H, d, *J* = 1.8 Hz, H-2'), 6.82 (1H, d, *J* = 8.4 Hz, H-5'), 7.66 (1H, dd, *J* = 8.4, 1.8 Hz, H-6'), 5.37 (1H, d, *J* = 7.8 Hz, H-1''), 3.17~3.66 (6H, m, H-2''-6''); ^13^C-NMR (DMSO-*d*_6_) see Table 2. By comparison with the literature data [32], compound **1** was identified as quercetin-3-*O-β*-d-galactopyranoside (hyperin).

*Compound* **2**. Yellow powder, ESI-MS (+ve ion mode): *m/z* 465.1 [M+H]^+^, ^1^H-NMR (DMSO-*d*_6_) δ: 6.19 (1H, d, *J* = 1.8 Hz, H-6), 6.40 (1H, d, *J* = 1.8 Hz, H-8), 7.58 (2H, m, H-2', 6'), 6.84 (1H, d, *J* = 9.0 Hz, H-5'), 5.46 (1H, d, *J* = 7.2 Hz, H-1''), 3.09~3.58 (6H, m, H-2''-6''). ^13^C-NMR (DMSO-*d*_6_) see Table 2. The ^1^H-NMR and ^13^C-NMR data were in agreement with that of quercetin-3-*O-β*-d-gluco-pyranoside (isoquercitrin) [33].

*Compound* **3**. Light yellow powder, ESI-MS (+ve ion mode): *m/z* 435.1 [M+H]^+^, ^1^H-NMR (DMSO-*d*_6_) δ: 6.20 (1H, d, *J* = 1.8 Hz, H-6), 6.40 (1H, d, *J* = 1.8 Hz, H-8), 7.54 (1H, d, *J* = 1.8 Hz, H-2'), 6.85 (1H, d, *J* = 8.4 Hz, H-5'), 7.57 (1H, dd, *J* = 8.4, 1.8 Hz, H-6'), 5.34 (1H, d, *J* = 7.2 Hz, H-1''), 3.20–3.63 (5H, m, H-2''-5''); ^13^C-NMR (DMSO-*d*_6_) see Table 2. The ^1^H-NMR and ^13^C-NMR data were in agreement with the quercetin-3-*O-β*-d-xylopyranoside (reynoutrin) data found in the literature [34].

*Compound* **4**. Yellow needle-like crystals; ESI-MS (+ve ion mode): *m/z* 435.1[M+H]^+^, ^1^H-NMR (DMSO-*d*_6_) δ: 6.20 (1H, d, *J* = 1.8 Hz, H-6), 6.40 (1H, d, *J* = 1.8 Hz, H-8), 7.51 (1H, d, *J* = 1.8 Hz, H-2'), 6.85 (1H, d, *J* = 8.4 Hz, H-5'), 7.66 (1H, dd, *J* = 8.4, 1.8 Hz, H-6'), 5.27 (1H, d, *J* = 5.4 Hz, H-1''), 3.20~3.75 (5H, m, H-2''-5''); ^13^C-NMR (150 MHz, DMSO-*d*_6_) see Table 2. The ^1^H-NMR and ^13^C-NMR data were in agreement with that of quercetin-3-*O-β*-d-arabinopyranoside [25].

*Compound* **5**. Yellow powder; ESI-MS (+ve ion mode): *m/z* 435.1 [M+H]^+^, ^1^H-NMR (DMSO-*d*_6_) *δ*: 6.20 (1H, d, *J* = 2.4 Hz, H-6), 6.41 (1H, d, *J* = 2.4 Hz, H-8), 7.48 (1H, d, *J* = 2.4 Hz, H-2'), 6.85 (1H, d, *J* = 8.4 Hz, H-5'), 7.55 (1H, dd, *J* = 8.4, 1.8 Hz, H-6'), 5.27 (1H, d, *J* = 5.4 Hz, H-1''), 3.28~3.72(5H, m, H-2''-5''). ^13^C-NMR (DMSO-*d*_6_) see Table 2. The ^1^H-NMR and ^13^C-NMR data were in agreement with the quercetin-3-*O-α*-L-arabinofuranoside data found in the literature [25].

*Compound* **6**. Brownish yellow powder; ESI-MS (+ive ion mode): *m/z* 573.2 [M+H]^+^, 595.2[M+Na]^+^, ESI-MS (‒ve ion mode): *m/z* 571.1[M-H]^−^. ^1^H-NMR (DMSO-*d*_6_) *δ*: 7.65 (2 H, dd, *J* = 7.2, 1.2 Hz, H-2', 6'), 7.46 (2 H, t, *J* = 7.2 Hz, H- 3', 5'), 7.55 (t, *J* = 7.2 Hz, H-4'), 2.00 (6 H, s, 3',5'-CH3), 4.63 (1 H, d, *J* = 7.8 Hz, H-1''), 6.96 ( 2 H, s, H-2''', 6'''); ^13^C-NMR (DMSO-*d*_6_) *δ*: 113.1 (C-1), 152.2 (C-2), 110.8 (C-3), 156.0 (C-4), 110.8 (C-5), 152.2 (C-6), 138.9 (C-1'), 128.7(C-2'), 128.2 (C-3'), 132.3 (C-4'), 128.2 (C-5'), 128.7 (C-6'), 196.9 (aglycone, C=O), 9.8 (3'-CH_3_), 9.8(5'-CH_3_), 104.1 (C-1''), 74.2 (C-2''), 76.1 (C-3''), 69.3 (C-4''), 73.4(C-5''), 62.7 (C-6''), 119.5 (C-1'''), 108.5(C-2'''), 145.3 (C-3'''), 138.4 (C-4'''), 145.3 (C-5'''), 108.5 (C-6'''). The ^1^H-NMR and ^13^C-NMR data were in agreement with that of 2,4,6-trihydroxy-3,5-dimethylbenzophenone 4-*O*-(6''-*O*-galloyl)-*β*-d-glucopyranoside data found in the literature [26].

**Table 2:** ^13^C-NMR Data of compounds **1**–**5**.

Atom	1	2	3	4	5
2	156.2	156.1	156.1	156.2	156.3
3	133.5	133.3	133.1	133.7	133.4
4	177.5	177.4	177.3	177.5	177.7
5	161.2	161.2	161.1	161.2	161.2
6	98.6	98.6	98.7	98.6	98.6
7	164.1	164.2	164.1	164.1	164.2
8	93.5	93.5	93.5	93.5	93.5
9	156.3	156.3	156.2	156.2	156.9
10	103.9	103.9	103.8	103.9	103.9
1'	121.1	121.1	120.9	120.9	120.9
2'	115.9	116.2	116.1	115.3	115.5
3'	144.8	144.8	144.8	144.9	145.0
4'	148.4	148.4	148.4	148.6	148.4
5'	115.1	115.2	115.3	115.7	115.5
6'	121.9	121.6	121.4	122.0	121.7
1''	101.8	100.9	101.7	101.4	107.8
2''	71.2	74.1	73.5	71.6	82.1
3''	73.2	76.5	75.9	70.7	76.9
4''	67.9	69.9	69.3	66.0	85.8
5''	75.8	77.5	66.0	64.2	60.6
6''	60.1	61.0			

## 4. Conclusions

This study demonstrates that HSCCC was a useful method for separating and isolating flavonoid glycosides and a benzophenone galloyl glycoside from the ethyl acetate extract from *P. guajava* leaves with a two-phase solvent system composed of *n*-hexane–ethyl acetate–methanol–water (0.7:4:0.8:4, v/v/v/v), confirming that HSCCC is an efficient technique to isolate and purificate pure bioactive compounds from natural products. Compared with the more extensive use of chromatography and preparative HPLC in natural product research, crude samples can be used in HSCCC and the sample size is larger. HSCCC has some shortcomings, such as poor efficiency and time consumption. As an emerging separation technology, the methods and techniques of HSCCC need further research.
